# Assessing Polymorph Stability and Phase Transitions at Finite Temperature: Integrating Crystal Structure Prediction, Lattice Dynamics, and Molecular Dynamics

**DOI:** 10.1021/acs.jctc.5c01387

**Published:** 2025-11-19

**Authors:** Gabriela B. Correa, Stefanos Konstantinopoulos, Benjamin I. Tan, Yong Zhang, Frederico W. Tavares, Claire S. Adjiman, Edward J. Maginn

**Affiliations:** † Department of Chemical and Biomolecular Engineering, 6111 University of Notre Dame, Notre Dame, Indiana 46556, United States; ‡ Chemical Engineering Program, 341391 Universidade Federal do Rio de Janeiro, Rio de Janeiro 21941-909, Brazil; § Department of Chemical Engineering, Sargent Centre for Process Systems Engineering, 4615 Imperial College London, London SW7 2AZ, United Kingdom

## Abstract

Determining finite-temperature polymorph stability and phase transitions remains a major challenge in crystal structure prediction (CSP). While static lattice energy methods, the zeroth-order CSP, may offer some initial insights, they neglect vibrational and entropic contributions that can influence stability under real-world conditions. Molecular dynamics (MD) approaches, on the other hand, can be used for free energy analysis across wide temperature ranges, but their computational cost limits their applicability to a small number of putative crystal structures. This work introduces a multistage protocol that combines CSP, harmonic approximation lattice dynamics (HA-LD), and MD to assess a larger number of structures than is possible with MD alone. Using tetracyanoethylene (TCNE) as a test case, we first carry out a zeroth-order CSP and then apply the relatively cheap HA-LD method to prescreen the 100 lowest-energy structures. Five thermodynamically relevant candidates are identified and advanced to detailed free energy analysis using the more expensive pseudosupercritical path (PSCP) method in MD. MD-PSCP captures the anharmonic and thermal expansion effects neglected in HA-LD, enabling excellent predictions of observed phase stability. The cubic–monoclinic enantiotropic transition of TCNE is predicted at 322 K, within the experimental range of 292–326 K, and the melting temperature of the monoclinic form is estimated with a deviation of less than 10 K from the experimental value. Our simulations also indicate a potentially more stable hypothetical form, whose viability requires further investigation. By combining CSP for structure generation, HA-LD for efficient prescreening, and MD-PSCP for enhanced predictive accuracy, our workflow overcomes the limitations of each method alone. This approach improves finite-temperature polymorph prediction and offers a practical framework for materials and pharmaceutical applications, where thermal effects are critical.

## Graphical abstract

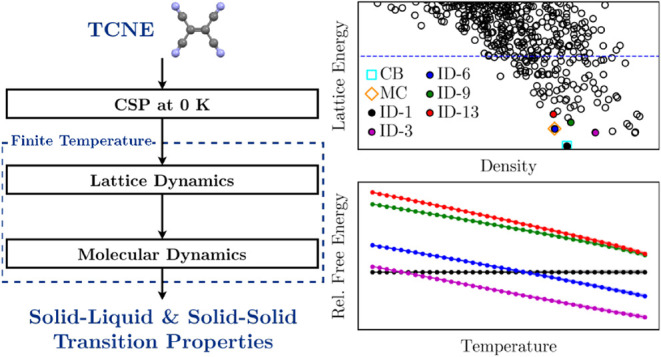

## 1 Introduction

Organic molecular crystals have a wide-range of applications in the pharmaceutical,^1^ agrochemical,^2^ and semiconductor^3^ industries (among others). Determining the molecular packing structure accurately is an essential prerequisite to understanding key properties of these solid materials.^4^ However, this endeavor is complicated by the fact that many organic compounds are polymorphic,^5–10^ capable of adopting multiple structures, each with a unique molecular arrangement. Polymorphs can exhibit markedly different physicochemical properties, such as solubility, bioavailability, and electronic characteristics. Historically, the failure to understand and mitigate the risks associated with polymorphism has led to severe repercussions arising from the unforeseen spontaneous transition between solid forms.^11–13^ As such, predicting the relative thermodynamic stability of polymorphs as a function of temperature (and pressure) has emerged as an important problem in solid-state chemistry. From this, one could envision determining complete phase diagrams for all observable solid forms.^14^

Crystal structure prediction (CSP) is a computational field of research that seeks to identify the most stable crystalline form of a compound, as well as any other metastable polymorphs that may be observed experimentally.^15,16^ From a thermodynamic perspective, at a given temperature (*T*) and pressure (*P*), the most stable crystalline form corresponds to the global minimum of the free energy landscape, and the remaining metastable forms represent local minima ranked in the order of ascending stabilities.^15^ While significant progress has been made in CSP methodologies, as exemplified through the Cambridge Crystallographic Data Center’s (CCDC’s) blind test series,^17–19^ CSP still faces outstanding challenges.^20,21^ Of particular relevance to this paper is the difficulty in determining how the relative free energies of the numerous crystal structures on a CSP landscape evolve with experimental conditions such as temperature. In fact, the most commonly practiced form of CSP today, termed CSP_0 (zeroth-order CSP),^20^ employs the approximation that the relative stability of predicted forms can be estimated from static lattice energy differences, rather than free energy differences.^20,21^ Although this approximation enables large-scale CSP polymorph screens,^16^ neglecting thermal and dynamical contributions to the free energy can lead to incorrect polymorph rankings under experimentally relevant conditions.^21–23^ Ignoring these effects is also suggested to contribute to the overprediction of observable polymorphs on the static lattice energy landscape.^24^ To address this limitation, various strategies have been developed to incorporate free energy corrections into CSP workflows, most often using lattice dynamics (LD) or molecular dynamics (MD).^16,18,19^ To balance the need for exploration and its cost, these relatively expensive corrections are typically applied to only a subset of likely structures identified from a previous CSP_0-type investigation.

LD theory^25^ provides a framework for evaluating the vibrational contributions to the free energy by means of phonon frequencies. In its simplest form, LD can be applied with the harmonic approximation (HA),^26^ which assumes perfect harmonic lattice vibrations, implying that the potential energy near a local minimum is parabolic. Under this approximation, the lattice geometry remains fixed at the lattice energy minimum configuration, restricting HA-LD to constant-volume (isochoric) calculations. HA-LD has seen increasing utilization in CSP.^17–19,22,27–32^ In the fifth CCDC blind test,^17^ no submissions attempted to use any form of LD. However, in the sixth^18^ and seventh^27^ blind tests, 6 and 5 groups (respectively) employed HA-LD as part of their CSP workflows. In most cases, the use of HA-LD led to more reliable rankings of the experimentally known forms. Hoja et al.^28^ and Firaha et al.^29^ have developed a workflow employing density functional theory-based HA-LD, which has shown good results for the test systems from the sixth blind test and for two pharmaceutical compounds. Nyman and Day^22^ have demonstrated that a non-negligible proportion of polymorphic molecules exhibit enantiotropic reranking of stability upon introduction of the vibrational contributions predicted with HA-LD.

The appeal of HA-LD can largely be attributed to its relatively good computational efficiency. However, the validity of the HA is expected to diminish with increasing temperature as its underpinning assumptions become less reliable. For instance, cell volumes have been observed to increase by 5–15% when comparing the predicted crystal geometries under 0 K static-condition and the experimentally reported crystals at finite temperature,^33,34^ thereby violating the isochoric-cell model. To accommodate cell expansion, LD can instead be performed with a quasi-harmonic approximation (QHA).^26^ The QHA extends the LD theory to account for isotropic^35^ and/or anisotropic^36^ thermal expansion by performing harmonic calculations over different unit cell volumes. At elevated temperatures, the assumption of a harmonic oscillator is also increasingly invalidated. To address this, LD theory can also be employed with an anharmonic approximation, (AA)^26^ wherein an anharmonic potential is used to describe vibrations.^28^ While the QHA and the AA are natural extensions of the HA, their added complexity can be prohibitive. For this reason, QHA- ^14,23,27,33^ and AA-LD^18,27^ (or their combination^28^) have seen less use with CSP, compared to HA-LD.

An alternative strategy to model the finite temperature behavior of crystals is through the use of MD. Unlike LD-based methods, MD natively models anharmonic motion and variable cell volumes,^35^ preserving its efficacy at elevated temperatures. Additionally, MD simulations are not limited to descriptions of the solid state and can be utilized to compute solid–liquid coexistence properties, such as melting points.^37^ In its simplest form, equilibrating crystal structures at finite temperature using MD can provide an assessment of crystal stability at real-world conditions.^27,38^ However, going further to assess relative polymorphic stability and/or explore free energy landscapes requires the use of more advanced MD techniques. To this end, enhanced sampling methods such as metadynamics^39–43^ and (crystal) adiabatic free energy dynamics,^38,44,45^ have been proposed and applied to a range of organic molecules. Common to both approaches is a reliance on collective variables to model the processes of interest. In practice, specifying appropriate sets of collective variables can pose a significant challenge to their routine use, particularly since they must be tailored to the system/process being studied.

Methods based on alchemical paths instead model a phase transition through a series of nonphysical intermediate states, which are accessed by gradually scaling the molecular interactions. Free energy differences along the alchemical path can then be estimated using statistical methods such as thermodynamic integration^46^ and multistate Bennett acceptance ratio (MBAR).^47^ Altogether, this permits the accurate assessment of free energy differences between states without direct transition sampling. The Einstein crystal method^48^ and the pseudosupercritical path (PSCP) method,^37,49,50^ are two variants of this alchemical approach, employing distinct paths. The former has been applied to pharmaceutical systems^51^ and the test molecules from the seventh blind test.^27^ Similarly, the PSCP method and its modifications have also been applied to a wide range of organic molecular systems,^24,35^ pharmaceutical and agrochemical-relevant compounds,^52–54^ and an organic salt.^55^ It is notable that the relative free energies between solid forms predicted with PSCP were shown to agree well with the values predicted by QHA-LD at 0–300 K.^35^ More recently, alchemical free-energy methods in CSP have been further expanded through deposition–sublimation paths based on MD with polarizable force fields, enabling the incorporation of finite-temperature and entropic effects in a polymorph search for Compound XXIII of the sixth blind text.^56^

While MD offers some clear advantages over LD methods, its application to finite temperature reranking of zeroth-order CSP landscapes is limited by two factors. First, MD methods usually rely on transferable, classical force fields that are not tailored to the solid state; this may cast some doubt on the accuracy of their predictions. Second, MD methods are costly, particularly when compared to HA-LD analysis. In the context of refining a zeroth-order CSP landscape, MD can be practicably applied to only a relatively small proportion of the landscape (usually not more than 30 structures^18,41,43,54^ ). This leaves a small margin for error in the CSP_0 predictions. Most attempts to alleviate these issues have involved employing multistage MD protocols. These include workflows applying MD equilibration followed by enhanced sampling methods^38,41–43^ as well as attempts to use the PSCP method with progressively higher-resolution simulation settings^52^ and different force field parametrizations.^27^

In this work, we aim to address the cost barrier associated with integrating MD techniques with zeroth-order CSP for the accurate prediction of solid phase-stability at finite temperature. We propose a multistage protocol that prescreens the CSP_0 landscape using HA-LD, followed by final free energy analysis with the PSCP method. The relatively low cost of HA-LD allows a larger portion of the CSP landscape to be screened than would be feasible with direct use of MD. The coarse assessment of finite temperature polymorph stability provided by this prescreening instills greater confidence that only the most-relevant structures are subject to the more expensive PSCP method. By leveraging two orthogonal and complementary techniques, we believe the proposed workflow can offer a better balance between cost and accuracy.

The remainder of this paper is structured as follows. In Section 2, the system of interest is introduced, followed by a description of the various computational methods. In Section 3, the proposed workflow is demonstrated. Importantly, leveraging the benefits of MD, we study both solid–solid (Section 3.4) and solid–liquid (Section 3.5) phase transitions in the final PSCP stage. Finally, concluding remarks are made in Section 4.

## 2 Methodology

### 2.1 Test System: Tetracyanoethylene

Tetracyanoethylene (TCNE, C_6_N_4_) has been chosen as the test system for this study. TCNE is a relatively rigid organic molecule comprising of 10 atoms, with its molecular structure shown in Figure 1.

**Figure 1:**
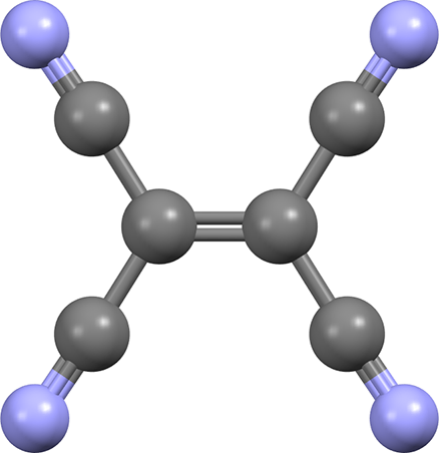
Tetracyanoethylene (TCNE, C_6_N_4_) molecule.

Four crystalline forms of TCNE have been reported in the literature.^57–59^ However, only the structures of the cubic (CB) and monoclinic (MC) forms have been resolved and can thus be considered in this work for the purpose of comparison to experimental data. Key crystallographic information is summarized in Table 1. These two forms emerge from distinct crystallization conditions^58^ and experimental results suggest that they are enantiotropically related.^58,60^ At low temperature the cubic form is purportedly more stable, while at elevated temperatures the monoclinic form becomes thermodynamically favored. A range of values for the solid–solid transition temperature have been reported between 292–326 K,^58,60^ depending on the measurement method. These studies also present conflicting reports regarding the reversibility of the phase transition.^58,60^ The unresolved polymorphs of TCNE are known to correspond to a hexagonal/trigonal form crystallized from methylene chloride/ethyl acetate solution at room temperature,^57^ and a high-pressure metastable form.^59^

**Table 1:** Experimentally Reported Forms of TCNE Crystals Studied in This Work^a^

Form	CSD Refcode	Space group	*Z*′/*Z*	Stability^58,60^
Cubic (CB)	TCYETY02 ^61^	*Im-3*	0.13/6	Stable at low *T*
Monoclinic (MC)	TCYETY04 ^62^	*P21/n*	0.50/2	Stable at high *T*

a The *Z*′ and *Z* values denote the number of molecules in the asymmetric unit cell and unit cell, respectively.

### 2.2 General Workflow

We propose a three-stage *in silico* workflow to evaluate the relative stability of TCNE crystals at finite temperature, as illustrated in Figure 2. This protocol integrates putative crystal structure generation by CSP, LD-based vibrational energy prescreening, and MD-based free energy analysis. Because the computational cost increases at each successive stage, only the most promising candidates (based on their relative stabilities) are carried forward. This preserves the balance between computational expense and accuracy over the entire workflow.

**Figure 2:**
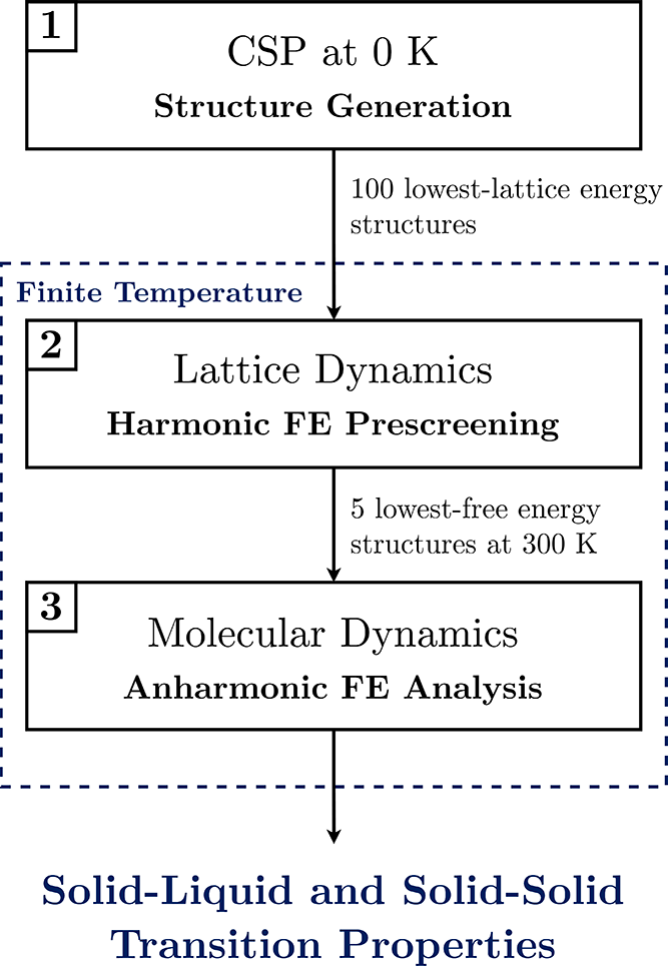
Workflow for evaluating thermodynamic stability of crystal structures under finite-temperature conditions. FE denotes free energy.

Mimicking “blind” conditions (i.e., assuming no experimental data are available), putative crystal structures are first proposed through a CSP study carried out at 0 K, static conditions. The state-of-the-art CSP procedure relies on a multistep process^16,18,19^ to balance the search breadth and accuracy. A global search is performed to exhaustively explore the crystal packing landscape and generate numerous plausible structures. The static lattice energies of these structures are evaluated by a relatively coarse energy model based on atomic-charges. The most promising global search candidates progress to a refinement step by a more-sophisticated energy model based on distributed multipole analysis.^63–66^

From this refined set, the 100 lowest-lattice energy structures are selected for finite temperature analysis and denoted ID-1 through ID-100, with ID-1 corresponding to the structure with the lowest lattice energy. The thermodynamic relevance of the 100 candidates are reassessed using lattice dynamics with a harmonic approximation, enabling efficient screening of a relatively large number of structures.^31,32^ By evaluating vibrational contributions to the Helmholtz free energy over a range of temperatures, dynamically unstable structures can be eliminated and a more realistic estimate of polymorph stability is obtained. The five most stable candidates at 300 K advance to the final stage for MD refinement. The PSCP method^37,49,50^ is employed to compute Gibbs free energy differences, capturing anharmonic and entropic contributions, that enable prediction of solid–liquid and solid–solid phase behavior under realistic thermodynamic conditions.

The following sections detail each computational stage and the associated methodologies.

### 2.3 Crystal Structure Prediction

The CSP study for TCNE is performed using the hybrid *ab initio*/empirical force field class of energy models. An extensive description of the energy model can be found elsewhere^67,68^ and only the salient details are discussed here.

In the chosen energy model, the static lattice energy of a crystal is decomposed as

1 $$U_{\mathrm{latt}}=\Delta U_{\mathrm{intra}}+U_{\mathrm{inter}}=\Delta U_{\mathrm{intra}}+U_{\mathrm{elec}}+U_{\mathrm{rd}}$$

where Δ*U*_intra_ and *U*_inter_ are the intramolecular and intermolecular contributions, respectively. The reference state for Δ*U*_intra_ (and by extension *U*_latt_) is a TCNE molecule *in vacuo* at its 0 K ground-state conformation. The intermolecular energy comprises independent contributions from electrostatic interactions, *U*_elec_, and from residual intermolecular interactions, *U*_rd_. It is often assumed that the leading components of the residual term are van der Waals-type repulsion and dispersion contributions (hence “rd”). All vibrational contributions and thermal effects are excluded from *U*_latt_.

The intramolecular contribution, Δ*U*_intra_, is given by

2 $$\Delta U_{\mathrm{intra}}=U_{\mathrm{intra}}^{\mathrm{crys}}-U_{\mathrm{intra}}^{\mathrm{vac}}$$

where $U_{\mathrm{intra}}^{\mathrm{crys}}$ and $U_{\mathrm{intra}}^{\mathrm{vac}}$ are conformational energies at the crystalline conformation and the ground-state conformation *in vacuo*, respectively. These conformational energies (and their associated conformations) are determined from appropriate *ab initio* calculations. The electrostatic interactions, *U*_elec_, are calculated from atomic-charges or distributed multipoles derived from the *ab initio* charge density for a given conformation. These electrostatic features are employed in a pairwise sum with long-range Ewald summation techniques^31,69,70^ to assess *U*_elec_ in the infinite lattice. It is assumed that *ab initio*-derived quantities can be approximated from quantum mechanical (QM) calculations on isolated molecules.

The residual intermolecular interactions, *U*_rd_, are associated with many-body effects and are poorly represented by isolated-molecule QM calculations. Instead, *U*_rd_ is described by a classical pair potential; in this work, a Buckingham pair potential is employed. The pairwise interaction energy between two atoms, *i* and *j*, with types *t* and *t*′ is given by

3 $$U_{\mathrm{rd},ij}=A_{tt^{\prime }}\exp\left(-\frac{r_{ij}}{B_{tt^{\prime }}}\right)-\frac{C_{tt^{\prime }}}{r_{ij}^{6}}$$

where *A_tt_*_′_, *B_tt′_*, and *C_tt_*_′_ are transferable potential parameters describing the *tt*′-interaction, and *r*_*ij*_ is the interatomic distance between *i* and *j*. To obtain *U*_rd_, a pairwise summation is performed over all unique pairs of interacting atoms (belonging to different molecules) within a finite real space cutoff distance.

In this work, the global search step is performed using CrystalPredictor (v2.4.3).^71–75^ At this step, we ascertain, based on chemical intuition and a computational analysis, that conformational changes between the TCNE molecule *in vacuo* and its crystalline forms are negligible (i.e., Δ*U*_intra_ ≈ 0), and treat the TCNE molecule as rigid. The ground state *in vacuo* TCNE conformation and associated CHelpG^76^ atomic-charges are determined at the PBE/6-31G­(d,p) level of theory using Gaussian 09.^77^ The derived conformation and charges are fixed throughout the global search. The FIT potential parametrization^78–83^ is used to describe *U*_rd_. All CrystalPredictor input settings are kept at their default values, including the *U*_rd_ and *U*_elec_ real space summation cutoffs, as well as the *U*_elec_ Ewald summation relative accuracy. CrystalPredictor uses a Sobol’ sequence^84^ to generate *Z*′ = 1 candidates in the 61 most common space groups, with space group sampling frequencies proportional to each group’s occurrence in the CSD.^85,86^ Five hundred thousand minimizations are carried out, following which local optima are clustered using the COMPACK^87^ algorithm to remove crystallographically equivalent structures.

All unique structures within +20.0 kJ/mol of the global search global minimum are advanced for refinement using CrystalOptimizer (v2.4.7),^88^ which interfaces with DMACRYS (v2.2.1.1).^89^ Although TCNE is unlikely to exhibit significant conformational flexibility, the subsequent lattice dynamics calculations require that the input structures (i.e., optimized structures from CrystalOptimizer) are minimized with respect to *all* atomic positions.^32^ In service of this, the TCNE molecule is treated with atomistic flexibility during the refinement step. To describe molecular flexibility, CrystalOptimizer employs local approximate models (LAMs), as detailed by Kazantsev et al.^88^ All QM calculations required for constructing LAMs are performed in Gaussian 09 ^77^ at the M06/6-31G­(d,p) level of theory, while distributed multipoles up to hexadecapoles are derived using Stone’s GDMA (v2.2) algorithm.^63–66^ The *U*_elec_ Ewald summation^90^ cutoffs in real and reciprocal space are determined using the default settings in DMACRYS. The FIT^78–83^ potential parametrization is used to describe *U*_rd_, with a real space cutoff of 30 Å. Upon completion of the refinement, the optimized structures are again subject to clustering using the COMPACK^87^ algorithm.

Following each CSP step (global search and refinement), CSP-predicted structures are identified as geometric matches to the known forms of TCNE if they return an RMSD_15_ value less than or equal to 0.50 Å, as determined by the COMPACK^87^ algorithm. RMSD_*N*_ scores generated by the COMPACK algorithm indicate the root-mean-square-deviation in atomic positions over an *N*-molecule cluster of nearest-neighbors, including a central reference molecule, thereby quantifying packing (dis)­similarity between crystal structures.

### 2.4 Lattice Dynamics

Lattice dynamics-based free energy calculations are performed on the 100 lowest-lattice energy structures from the CSP refinement (denoted ID-1 through 100). These calculations are conducted within the harmonic approximation, which offers a computationally efficient and low-cost approach for prescreening candidate structures. The formalism for lattice dynamics was introduced in the pioneering work of Born and Huang,^25^ and details of its extension to the atomistic treatment of organic molecular crystals can be found in our previous work.^31,32^

In the harmonic approximation of LD theory, the Helmholtz free energy of a crystal at temperature *T* is given as the sum of the static lattice energy and the vibrational contribution to free energy (*A*_vib_),^22^

4 $$A\left(T\right)=U_{\mathrm{latt}}+A_{\mathrm{vib}}\left(T\right)$$

The vibrational contribution can be further broken down as

5 $$A_{\mathrm{vib}}\left(T\right)=U_{\mathrm{ZPE}}+U_{\mathrm{thermal}}\left(T\right)-TS$$

where *U*_ZPE_ describes the zero-point energy (ZPE) arising from the QM treatment of the harmonic oscillator, while *U*_thermal_ and *TS* are the contributions from thermal energy and entropy, respectively.

Collectively, *A*_vib_(*T*) can be expressed as^22^

6 $$A_{\mathrm{vib}}\left(T\right)=\underset{k}{\sum }\overset{3N}{\underset{n=1}{\sum }}\frac{1}{2}\hbar \omega_{n}\left(k\right)+\underset{k}{\sum }\overset{3N}{\underset{n=1}{\sum }}k_{B}T\ln\left[1-\exp\left(\frac{-\hbar \omega_{n}(k)}{k_{B}T}\right)\right]$$

where *k*_B_ and ℏ are the Boltzmann constant and reduced Planck constant, respectively. ***k*** is the reciprocal space wave vector, while *n* is the vibrational mode index. For a system with *N* atoms in the unit cell, there are 3*N* modes of vibration associated with a phonon angular frequency (ω_*n*_). In eq 6, the first term on the right-hand side corresponds to the *U*_ZPE_ contribution, while the second term is the combined contribution of *U*_thermal_(*T*)–*TS*. From this, it can be inferred that at *T* = 0 K, only the ZPE contributes to *A*_vib_. Independent expressions for *U*_thermal_(*T*) and *TS* can be derived using the isochoric heat capacity and entropy;^22,32^ here we directly employ eq 6 to determine *A*_vib_(*T*).

An important aspect when calculating *A*_vib_ is determining values for ω_*n*_. To this end, the dynamical matrix is first constructed by taking a mass-weighted Fourier transform of the force constant matrix. The force constant matrix is the Hessian matrix derived from the potential energy with respect to atomic positions. By diagonalization, the dynamical matrix’s eigenvalues (λ_*n*_) can be found, from which each ω_*n*_ is given as the square-root of the corresponding λ_*n*_. As the dynamical matrix is Hermitian, it yields real eigenvalues that have a physical interpretation. A structure is stable on the free energy landscape if its dynamical matrix is positive-definite (i.e., λ_*n*_ > 0, ∀ *n*), such that all ω_*n*_ are positive real values. This requires the preceding CSP refinement to have converged tightly to a minimum relative to *all* atomic positions, with a positive-definite force constant matrix. Conversely, negative eigenvalues will yield imaginary frequencies indicating unstable structures on the free energy landscape.

In our calculations, *A*(*T*) is evaluated at temperatures ranging from 0 to 400 K in 20 K increments. These are single-point free energy evaluations, meaning the geometry of a given structure is held fixed, as determined during the CSP refinement step. To facilitate analysis, we define the relative Helmholtz free energy between structures S_*j*_ and S_*i*_ as

7 $$\Delta A_{S_{i}\to S_{j}}\left(T\right)=A_{S_{j}}\left(T\right)-A_{S_{i}}\left(T\right)$$

where $A_{S_{i}}$ and $A_{S_{j}}$ denote the Helmholtz free energy of ID-*i* and ID-*j* solid forms, respectively.

The CrystalDynamics (v1.1) program^31,32^ is used to perform the finite temperature analysis based on the HA of lattice dynamics. The CSP refinement energy model must be retained during lattice dynamics calculations as introducing a new model may lead to the Hessian matrix no longer being positive definite, thereby violating a core requirement of the harmonic approximation. To this end, CrystalDynamics is interfaced with Gaussian 09 ^77^ to perform QM calculations at the M06/6–31G­(d,p) level of theory. This is used to evaluate the derivatives of Δ*U*_intra_ with respect to atomic coordinates. From the QM calculations, distributed multipoles up to hexadecapoles are derived using GDMA (v2.2)^63–66^ and employed in determining the analytical derivatives of *U*_elec_. To achieve consistency between the CSP-refinement model and the LD model, the *U*_elec_ Ewald summation performed in CrystalDynamics employs a real space cutoff of 15.97 Å, a reciprocal space cutoff of 1.05 Å^–1^, and an Ewald convergence parameter of 0.08. Finally, analytical expressions for the derivatives of *U*_rd_ have been derived^32^ and these are evaluated using the FIT^78–83^ pair potential parameters with a real space cutoff of 30 Å.

For molecular solids, external vibrational modes arise from the collective motion of molecules as rigid entities. These vibrational modes tend to have low frequencies and therefore contribute significantly to *A*_vib_. To account for these vibrational modes during lattice dynamics calculations, adequately large supercells must be employed. To this end, based on the chosen intermolecular cutoff distances and the target crystal structure, CrystalDynamics automatically sizes an *n*_sc_ × *n*_sc_ × *n*_sc_ supercell, wherein *n*_sc_ is the minimum integer value exceeding the ratio of cutoff distance to shortest-unit-cell-length. A more detailed description of this supercell sizing protocol is provided in our previous work.^31,32^

### 2.5 Molecular Dynamics

Molecular dynamics-based free energy calculations are performed on the five lowest free energy crystal structures identified through the lattice dynamics screening at 300 K. The calculations employ the PSCP method and the MBAR estimator to ensure statistical robustness, as detailed in our previous work.^37,55^ These results form the basis for assessing phase stability and transition properties.

MD simulations are carried out using the LAMMPS package^91^ in both isothermal–isobaric (NPT) and canonical (NVT) ensembles, applying periodic boundary conditions and a time step of 1 fs. Long-range electrostatic interactions are treated using the particle–particle particle-mesh method^92^ with a cutoff of 12 Å and a relative error tolerance of 1 × 10^–4^. Temperature and pressure control are achieved using the Nosé-Hoover thermostat^93^ and the Martyna–Tobias–Klein barostat,^94^ with characteristic times of 100 fs and 1000 fs, respectively. During NPT simulations, pressure is maintained at 1 atm, allowing anisotropic cell changes for crystal phases and isotropic volume changes for the liquid phase.

A refined version of the all-atom Optimized Potentials for Liquid Simulations (OPLS) force field^95^ is used to model the interatomic interactions. The default OPLS atomic partial charges are replaced with values obtained using the restrained electrostatic potential (RESP) method,^96^ fitted to the electrostatic potential surface of the optimized molecular geometry at the B3LYP/6-311++G­(d,p) level of theory. The nominal intramolecular bonds and angles are also extracted from this optimized geometry and used in place of the original OPLS values. Other parameters are taken directly from OPLS. QM optimization is carried out using Gaussian 09.^77^ All force field parameters are summarized in Figure S1 and Table S1 of the Supporting Information.

Initial crystal configurations are generated by replicating the unit cells of each structure to produce simulation boxes with dimensions at least 2.5 times the interaction cutoff. The supercells constructed in this manner contain between 144 and 200 molecules, sufficient to mitigate finite-size effects in molecular crystals, per the findings of Dybeck et al.^35,97^ The liquid phase is modeled using 200 randomly distributed molecules in a cubic simulation box. All systems undergo an initial 5 ns equilibration in the NPT ensemble, from which equilibrium densities are determined based on the final 3 ns of each trajectory.

The solid–liquid (*T*_m_) and solid–solid (*T*_ss_) phase transition temperatures are determined by identifying the temperature at which the Gibbs free energy difference between phases becomes zero. This difference is computed as^52,98^

8 $$\Delta G_{p1\to p2}\left(T\right)=k_{B}T\left[\Delta f_{p1\to p2}\left(T\right)-\Delta f_{p1\to p2}\left(T_{\mathrm{ref}}\right)\right]+\frac{T}{T_{\mathrm{ref}}}\Delta G_{p1\to p2}\left(T_{\mathrm{ref}}\right)$$

where *T*_ref_ is the reference temperature, p1 and p2 denote the initial and final phases-either liquid (L) or solid polymorphs (S_1_, ..., S_*j*_). The reduced Gibbs free energies, Δ*f*_p1→p2_, are obtained from NPT simulations spanning 110–490 K in 10 K increments. The reference Gibbs free energy, Δ*G*_p1→p2_(*T*_ref_), is computed from NVT simulations in PSCP cycles at *T*_ref_. A *T*_ref_ of 350 K is selected as an approximate midpoint of the analyzed temperature range.

In the PSCP method, Helmholtz free energies are first computed by progressively transforming one phase into another through a series of nonphysical intermediate states. These Helmholtz free energies are then converted into Gibbs free energies using pressure–volume corrections. PSCP cycles for both solid–liquid (melting) and solid–solid transitions are presented in Figure 3. Melting is modeled as a three-step process: (i) gradual reduction of intermolecular interactions in the solid phase to yield a dense weak fluid (DWF), (ii) expansion of the DWF to match the liquid density, producing a weak fluid (WF), and (iii) reintroduction of full interactions to obtain the liquid phase. Solid–solid transitions follow a closely related four-step scheme. Each polymorph is independently converted into a DWF, followed by expansion and unification into a common WF.

**Figure 3:**
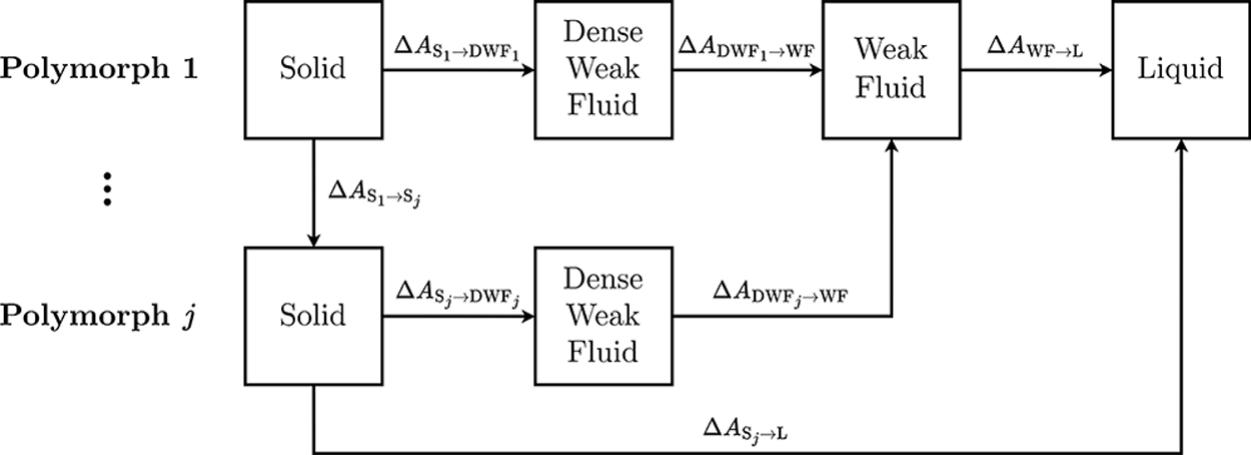
A schematic of the PSCP method for solid–liquid and solid–solid transformations.

Interaction scaling in the S_*j*_ → DWF_*j*_ and WF → L steps is controlled by a coupling parameter, λ. In the S_*j*_ → DWF_*j*_ step, we use 40 intermediate λ values equally spaced across the full range (λ = 0 to 1), and additional values concentrated between λ = 0.8 and 0.9 to improve sampling resolution in a region of steep energy change. The WF → L step is also performed by increasing λ from 0 to 1, using 20 equally spaced values. The DWF → WF expansion is carried out by gradually increasing the volume from that of the solid to that of the liquid, using 20 equally spaced volume states. At each intermediate state, simulations are run for 5 ns and the final 3 ns are used for analysis. Gaussian potential restraints are applied to all 10 atoms of the TCNE molecule. To evaluate the convergence of the calculations, the well depths of the Gaussian potential are tested at two values, 20.92 and 41.84 kJ/mol. These values are selected by fitting the probability distribution of three-dimensional harmonic oscillators observed in NVT simulations of the crystal phases.

The total Helmholtz free energies at *T*_ref_ are computed from the contribution of all relevant transformation steps as^55^

9a $$\Delta A_{S_{j}\to L}\left(T_{\mathrm{ref}}\right)=\Delta A_{S_{j}\to {\mathrm{DWF}}_{j}}\left(T_{\mathrm{ref}}\right)+\Delta A_{{\mathrm{DWF}}_{j}\to \mathrm{WF}}\left(T_{\mathrm{ref}}\right)+\Delta A_{\mathrm{WF}\to L}\left(T_{\mathrm{ref}}\right)$$

9b $$\Delta A_{S_{1}\to S_{j}}\left(T_{\mathrm{ref}}\right)=\Delta A_{S_{1}\to {\mathrm{DWF}}_{1}}\left(T_{\mathrm{ref}}\right)+\Delta A_{{\mathrm{DWF}}_{1}\to \mathrm{WF}}\left(T_{\mathrm{ref}}\right)-\Delta A_{S_{j}\to {\mathrm{DWF}}_{j}}\left(T_{\mathrm{ref}}\right)-\Delta A_{{\mathrm{DWF}}_{j}\to \mathrm{WF}}\left(T_{\mathrm{ref}}\right)$$

The corresponding Gibbs free energies are obtained using

10 $$\Delta G_{p1\to p2}\left(T_{\mathrm{ref}}\right)=\Delta A_{p1\to p2}\left(T_{\mathrm{ref}}\right)+P\Delta V_{p1\to p2}\left(T_{\mathrm{ref}}\right)$$

At near-ambient pressures, the pressure–volume term of the Gibbs free energy is typically negligible (less than 0.01 kJ/mol), justifying the approximation Δ*G* ≈ Δ*A*.^99^ At elevated pressures (e.g., > 1 GPa), the pressure–volume term can become significant and pressure-induced solid–solid phase transitions may occur;^59,100,101^ however, this is beyond the scope of this work.

Additional properties, such as enthalpy and heat capacity differences, are also evaluated at the transition temperatures. Enthalpy changes are obtained from NPT simulations by computing ensemble-averaged enthalpies for each phase, using $\Delta H_{m}=\langle H\rangle_{L}-\langle H\rangle_{S_{j}}$ and $\Delta H_{\mathrm{ss}}=\langle H\rangle_{S_{j}}-\langle H\rangle_{S_{1}}$ . The heat capacity differences during melting are calculated from the temperature derivatives of the enthalpy changes, i.e., $\Delta C_{p,m}={\partial \langle H\rangle_{L}/\partial T|}_{P}-{\partial \langle H\rangle_{S_{j}}/\partial T|}_{P}$ .

The statistical uncertainty in the Gibbs free energy difference is obtained by error propagation from eq 8 as

11 $${\left(\delta \frac{\Delta G}{k_{B}T}\right)}^{2}=(\delta \Delta f{)}^{2}+(\delta \Delta f_{\mathrm{ref}}{)}^{2}+{\left(\delta \frac{\Delta G_{\mathrm{ref}}}{k_{B}T_{\mathrm{ref}}}\right)}^{2}$$

where each term is provided by the MBAR estimator within 95% confidence intervals. The overall PSCP uncertainty is evaluated by propagating the statistical errors of all stages at the reference temperature. Uncertainties in transition temperatures are determined from the intersection of the Δ*G*_p1→p2_ = 0 line with the confidence bands, and the same procedure is applied to estimate the uncertainties in enthalpy, entropy, and heat capacity differences.

## 3 Results and Discussion

### 3.1 CSP Structure Generation

The global search step, which involves 500,000 minimizations, yields 1188 unique structures within a +20 kJ/mol energy window of the global minimum. The corresponding global search landscape is presented in Figure 4a. As shown in Table 2, good geometry matches to both forms of TCNE are found with RMSD_15_ < 0.40 Å, validating the rigid-body approximation. Due to the restriction to integer *Z*′ in CrystalPredictor, CrystalOptimizer, and CrystalDynamics, the CSP-predicted matches emerge in lower symmetry space groups compared to their experimentally reported counterparts. Specifically, matches to the cubic and monoclinic forms are found in the *R3* and *Pn* space groups, respectively. The match to the cubic form is ranked fourth on the global search landscape, with a relative energy to the global minimum $\left(\Delta U_{\mathrm{latt}}^{\mathrm{GM}}\right)$ of +1.368 kJ/mol. The match to the monoclinic form is ranked 12th and has a slightly higher $\Delta U_{\mathrm{latt}}^{\mathrm{GM}}=+2.914$ kJ/mol. Given that the central goal of the global search is to identify all putative crystal structures (within a reasonable $\Delta U_{\mathrm{latt}}^{\mathrm{GM}}$ ), these results are adequate for advancing to the refinement step.

**Figure 4:**
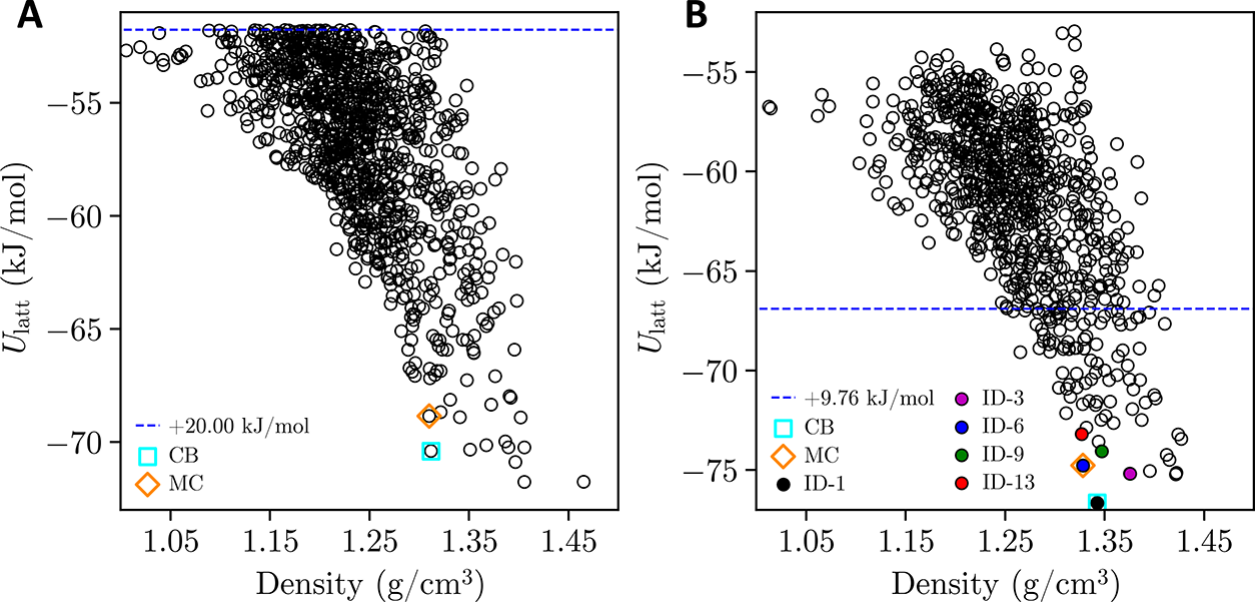
Postclustering *U*_latt_ CSP landscape from (a) the global search step, and (b) the refinement step. Matches to the cubic (CB) and monoclinic (MC) forms are indicated by the unfilled cyan square and unfilled orange diamond, respectively. In (b), ID-1 (black), ID-3 (purple), ID-6 (blue), ID-9 (green), and ID-13 (red) are denoted by filled circles. These five structures were ultimately chosen for MD analysis, as discussed below.

**Table 2:** Summary of Matches to the Known TCNE Polymorphs at Each Step of the Static Lattice Energy CSP Study

	Global Search		Refinement	
	CB	MC	CB	MC
RMSD_15_ (Å)	0.118	0.398	0.066	0.301
$\Delta U_{\mathrm{latt}}^{\mathrm{GM}}$ (kJ/mol)	+1.368	+2.914	+0.000	+1.883
Rank	4th	12th	1st	6th

In the refinement step, the lattice energies of the 1188 global search structures are reminimized with atomistic flexibility and a distributed multipole electrostatic model (in contrast to the earlier rigid-molecule, atomic-charge model). This reduces the static lattice energy landscape to 820 unique minima, as shown in Figure 4b. Geometric matches to the known forms of TCNE exhibit better agreement (lower RMSD_15_, as seen in Table 2) with their corresponding experimental reference geometries. Critically, the relative stabilities of the predicted matches also improve following the refinement. The cubic form becomes the global minimum, while the monoclinic form is only $\Delta U_{\mathrm{latt}}^{\mathrm{GM}}=+1.883$ kJ/mol higher in energy at rank 6. The enhanced CSP predictions (in crystal geometries and energies) may be attributable to several factors: the higher level of QM theory (M06 versus PBE), the more-sophisticated electrostatic model (distributed multipoles versus atomic-charges), and the greater permitted conformational flexibility (atomistic versus rigid body).

Ultimately, the results from the refinement step are consistent with the cubic form being the most stable polymorph at low temperatures (e.g., 0 K static conditions). Moreover, the predicted energy difference between the cubic and monoclinic forms aligns with the expected static lattice energy difference for enantiotropic polymorphs.^22,23^

### 3.2 Lattice Dynamics Prescreening

The 100 lowest-energy structures (denoted ID-1 through 100) progress for lattice dynamics prescreening, spanning an energy window of up to +9.76 kJ/mol on the refinement landscape. Of these 100 structures, only 70 exhibit positive phonon frequencies along all sampled ***k*** points, and thus emerge as stable on the free energy landscape. The remaining 30 structures exhibit imaginary frequencies and are eliminated from the landscape. The Helmholtz free energies relative to that of ID-1 for the temperature range 0–400 K are presented in Figure 5a for the 70 stable solid forms.

**Figure 5:**
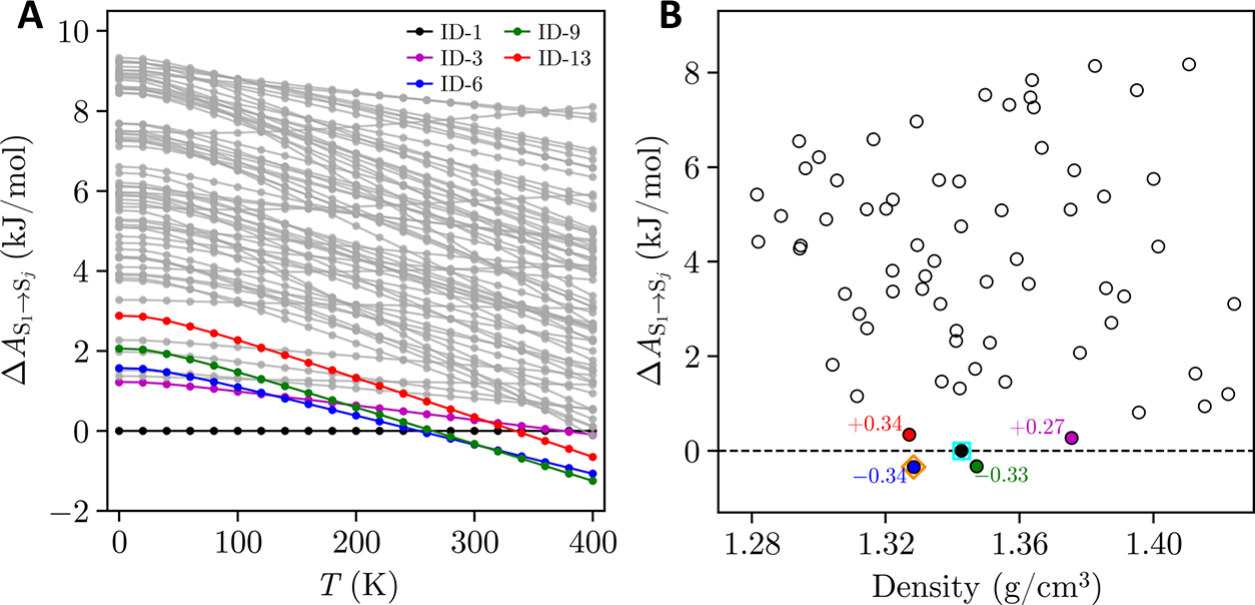
Relative Helmholtz free energy landscape obtained by HA-LD for the 70 CSP-predicted structures with positive phonon frequencies: (a) between 0–400 K, and (b) at 300 K. ID-1 (black), ID-3 (purple), ID-6 (blue), ID-9 (green), and ID-13 (red) are colored accordingly. In (b), matches to the CB and MC forms are indicated by the unfilled cyan square and unfilled orange diamond respectively, while ID-3 (purple), ID-6 (blue), ID-9 (green), and ID-13 (red) are labeled with their relative energies, compared to ID-1 (dashed black line).

Only the ZPE vibrational term contributes to the Helmholtz free energies at 0 K. Comparing the matches to the cubic (ID-1) and monoclinic (ID-6) forms, the absolute difference in the *U*_ZPE_ contributions is 0.361 kJ/mol. This value is consistent with results from Nyman and Day,^22^ who suggested that an overwhelming majority of polymorphic pairs (∼95%) exhibit |Δ*U*_ZPE_| < 0.33 kJ/mol. Ultimately, the *U*_ZPE_ term only has minor influence over relative free energies^22,31^ and relative rankings do not change significantly on the 0 K Helmholtz free energy landscape (accounting for structures that are eliminated due to imaginary phonon frequencies). The 0 K Helmholtz free energy landscape is presented in Figure S2.

Given that the enantiotropic transition between the cubic and monoclinic forms is expected to occur between 292–326 K,^58,60^ we focus our analysis on the 300 K Helmholtz free energy landscape (Figure 5b). At this temperature, a significant reordering of relative stabilities is observed among the predicted TCNE crystal structures, compared to their static lattice energy rankings. The five lowest-energy structures on the 300 K Helmholtz free energy landscape are ID-1, ID-3, ID-6, ID-9, and ID-13. ID-1 (i.e., the cubic form), which was the global minimum of the CSP refinement step, is instead ranked third on the 300 K Helmholtz free energy landscape. ID-6 (i.e., the monoclinic form) becomes the global minimum, correctly suggesting that an enantiotropic transition would be observed between the cubic and monoclinic forms. ID-9 is ranked second at this temperature, suggesting that this structure is also more stable than the cubic form (ID-1) at 300 K. Finally, ID-3 and ID-13 remain less stable than ID-1, but are in close thermodynamic competition with relative free energy gaps <0.35 kJ/mol.

Although this LD analysis correctly reflects the cubic–monoclinic enantiotropic relationship, it is not without flaws. First, from Figure 5a, the transition from ID-1 to ID-6 is predicted to occur at ∼250 K. While this is a reasonable estimate for *T*_ss_,^102^ it is still ∼40 K in error from the experimentally reported transition temperatures.^58,60^ Moreover, from the LD trajectories, a crossover from ID-6 to ID-9 occurs ∼320 K, suggesting that a transition should be observed before melting, yet this has not been reported experimentally. It is possible that the ID-6 (monoclinic) form is kinetically trapped (and/or ID-9 is kinetically inaccessible), precluding this experimental observation. However, it is also likely that this result simply reflects the diminishing reliability of the harmonic approximation as temperature increases.

In light of this, within our proposed protocol, we do not attempt to draw conclusions about TCNE’s finite temperature phase stability from the HA-LD analysis. Instead, HA-LD is used solely as a preliminary (finite temperature) reranking of the static lattice energy landscape. ID-1, ID-3, ID-6, ID-9, and ID-13 are the 5 most-stable structures over a fairly broad range of temperatures (240–390 K). Moreover, at 300 K, the sixth ranked structure (ID-5) is an additional +0.47 kJ/mol away from ID-13 (equivalent to +0.81 kJ/mol from ID-1 and +1.15 kJ/mol from ID-6). Together, these results suggest that HA-LD offers adequate accuracy, with margin for error, to identify the thermodynamically competitive structures at finite temperature, even if the details of their relative stabilities may not be exactly correct. Critically, the relatively low computational cost of HA-LD enables this prescreening to be performed over a larger portion of the static lattice energy landscape than would be practicable with the MD protocol alone. This serves as a valuable intermediate stage that reduces the risk of erroneously rejecting structures from the zeroth-order CSP to the final finite temperature reranking.

### 3.3 Molecular Dynamics Initial Assessment

From the HA-LD prescreening, ID-1, ID-3, ID-6, ID-9, and ID-13 progress to the final MD analysis stage; their packing representations as obtained from the CSP landscape at 0 K are shown in Figure 6. As described in Section 2, MD simulations of these solid forms use supercells with dimensions at least 2.5 times the chosen interaction cutoffs. Specifically, the replication schemes for each structure are as follows: 2 × 2 × 4 for ID-1 (144 molecules), 4 × 5 × 5 for ID-3 and ID-6 (200 molecules each), and 3 × 5 × 3 for ID-9 and ID-13 (180 molecules each). For comparison, the CSD entries for the cubic and monoclinic phases listed in Table 1 are also constructed using 3 × 3 × 3 (162 molecules) and 4 × 5 × 4 (160 molecules) replications, respectively.

**Figure 6:**
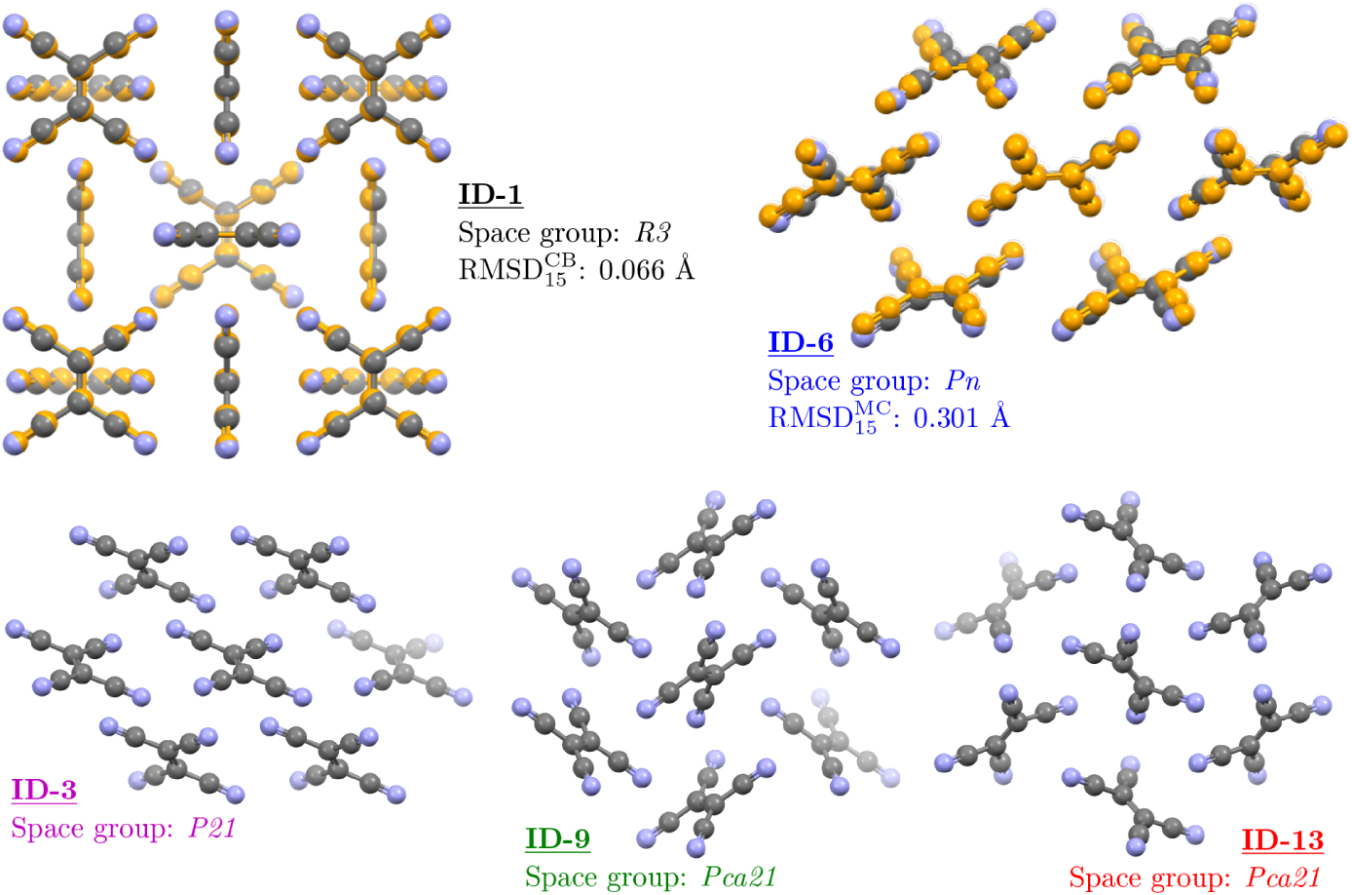
Visualization of all whole TCNE molecules within a 5 Å packing sphere for the five lowest-energy TCNE structures on the 300 K Helmholtz free energy landscape. The experimental geometries for the CB and MC forms are overlaid, using COMPACK, in orange over ID-1 and ID-6 respectively, illustrating their geometric agreement.

For each crystal structure, the Gibbs free energy at 0 K, which is identical to the enthalpy, is obtained by minimization using the modified OPLS force field. During this initial assessment, the lattice parameters of each structure are fixed at the CSP-predicted values, while all intramolecular degrees of freedom are fully relaxed to ensure internal consistency with the force field. In Table 3, these relative Gibbs free energies are compared to the corresponding relative Helmholtz free energies predicted by HA-LD. Free energies calculated via both methods are directly comparable under the studied conditions, as Δ*G* ≈ Δ*A*. The HA-LD calculations correspond to single-point evaluations performed at the CSP-predicted geometries, where both lattice parameters and atomic positions are kept fixed. The HA-LD results are expected to serve as a useful benchmark at 0 K, since anharmonic effects are minimal at low temperatures^27^ and the energy model used in LD is specifically parametrized for the solid state.

**Table 3:** Predicted Lattice Parameters, Density, and the 0 K Free Energies (Relative to ID-1) Calculated by Lattice Dynamics (HA-LD) and Molecular Mechanics (MM) Constrained Relaxation with the Modified OPLS Force Field^a^

								$\Delta E_{S_{1}\to S_{j}}$ (kJ/mol)	
Structure	*a* (Å)	*b* (Å)	*c* (Å)	α (deg)	β (deg)	γ (deg)	Density (g/cm^3^)	HA-LD	MM
ID-1	13.905	13.905	8.515	90.0	90.0	120.0	1.343	0.00	0.00
ID-3	7.830	6.128	6.522	90.0	81.2	90.0	1.376	+1.22	+0.84
ID-6	7.999	6.020	6.742	90.0	80.5	90.0	1.328	+1.57	+2.64
ID-9	10.311	6.191	9.893	90.0	90.0	90.0	1.347	+2.06	+4.73
ID-13	10.601	5.939	10.183	90.0	90.0	90.0	1.327	+2.88	+5.98

a *E* refers to Helmholtz free energy (*A*) for HA-LD and Gibbs free energy (*G*) for MM.

Both methods yield consistent stability rankings at 0 K, with ID-1 as the most stable polymorph and ID-13 as the least stable among the five structures. However, the relative free energies from the modified OPLS force field are generally higher than those from LD, except for ID-3. Expecting quantitative agreement between the HA-LD and OPLS predictions is unrealistic given that the underlying energy models are distinct. Broadly speaking, the OPLS force field uses atomic-charges and has been parametrized to liquid-phase data, while the LD model relies on distributed multipoles and a solid-state tailored pair potential. Bearing these model differences in mind, the qualitative agreement between the two model predictions is commendable and provides some credibility for the selected MD model.

MD simulations at 295 K are performed to equilibrate the five CSP-predicted structures, along with the experimentally reported cubic and monoclinic forms. The MD-equilibrated lattice parameters and the corresponding RMSD_*N*_ scores are presented in Table 4. For each system, the RMSD_*N*_ is calculated between the reference structure and the lowest potential-energy MD-equilibrated structure, using all *N* molecules available in their respective supercells. For the experimental structures (i.e., “CB” and “MC”), comparisons are made against their reported structures; for the others (i.e., “ID-*j*”), the CSP-predicted geometries serve as the reference. In all cases, COMPACK successfully overlays all *N* molecules, indicating that each structure remains stable at 295 K within the MD landscape.

**Table 4:** Lattice Parameters and Densities in the Reference/Initial Structures and Following MD-Equilibration at 295 K^a^

Structure	*a* (Å)	*b* (Å)	*c* (Å)	α (deg)	β (deg)	γ (deg)	Density (g/cm^3^)	RMSD_*N*_ (Å)
	Reference structures (Exp.: 295 K and CSP: 0 K)							
CB (Exp.)	9.736	9.736	9.736	90.0	90.0	90.0	1.383	N/A
MC (Exp.)	7.492	6.214	6.999	90.0	97.2	90.0	1.316	N/A
ID-*j* (CSP)	CSP-predicted crystal structures in Table 3							N/A
	MD-equilibrated structures (295 K)							
CB	9.958	9.959	9.959	90.0	90.0	90.0	1.292	0.471 (162)
MC	8.387	5.971	6.767	90.0	99.8	90.0	1.274	1.168 (160)
ID-1	14.081	14.081	8.624	90.0	90.0	120.0	1.293	0.376 (144)
ID-3	7.295	6.172	7.218	90.0	85.5	90.0	1.313	1.387 (200)
ID-6	8.384	5.972	6.769	90.0	80.2	90.0	1.274	0.587 (200)
ID-9	10.315	6.309	10.273	90.0	90.0	90.0	1.273	0.601 (180)
ID-13	10.777	5.883	10.722	90.0	90.0	90.0	1.252	0.648 (180)

a Statistical uncertainties are 2% of the reported values. RMSD_N_ in atomic positions is reported for each MD supercell, relative to their reference geometries. Values of *N* are given in parentheses.

The equilibrated structures for the cubic and monoclinic forms exhibit lattice parameters that closely match the experimental values; the resulting MD densities are lower than the experimental values by less than 7%. Among the CSP-predicted forms, all five exhibit a 3–6% increase in cell volume relative to the CSP-predicted reference. This aligns with the expected cell expansion associated with thermal effects.^33^ Readers are reminded that this moderate increase in cell volume is neglected in HA-LD, as isochoric behavior is assumed, which again justifies our use of HA-LD solely as a prescreening tool. Notably, ID-1 (cubic) and ID-6 (monoclinic) reach equilibrated densities identical to their simulated experimental counterparts, suggesting convergence to the same MD-stable structures. Additional analysis is presented in Figure S3, which shows density and enthalpy profiles over 110–490 K, confirming the crystals’ stability beyond 295 K during MD simulations. In Figure S4, structural calculations verify that the modified OPLS force field captures TCNE’s rigid, near-planar geometry, with ID-1 displaying distinct C–C–C angles consistent with experimental cubic–monoclinic differences.

These initial comparisons of MD predictions to HA-LD energies and experimental geometries suggest that the chosen MD model is adequate for modeling TCNE. This also highlights that in the absence of experimental data, HA-LD prescreening can offer a source of reference data to cross-check the MD model, at low computational cost. Only low-temperature HA-LD data should be used for MD model validation, to temper the limitations of HD-LA.^27^

### 3.4 Solid–Solid Transition Predictions

The five CSP-predicted TCNE polymorphs in Figure 6 are simulated using MD to compute their Gibbs free energies relative to ID-1 at finite temperature, as depicted in Figure 7. These values are obtained between 110–490 K using eq 8, based on the PSCP method performed at 350 K. Detailed free energy estimates at each step of the PSCP protocol are presented in Table S2, including a convergence analysis performed using two different well depths for the Gaussian potential.

**Figure 7:**
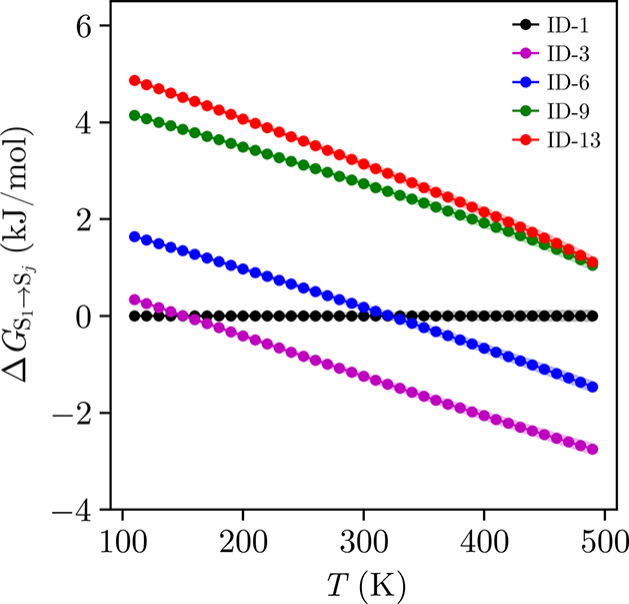
Relative Gibbs free energies as a function of temperature, calculated via PSCP-based MD simulations for ID-1 (black), ID-3 (purple), ID-6 (blue), ID-9 (green), and ID-13 (red). The shaded regions indicate the associated uncertainties of the simulation results.

Among the CSP-identified experimental structures, ID-1 is more stable up to 322 K. Above this temperature, ID-6 becomes more stable, exhibiting the lower Gibbs free energy. This result aligns with experimental observations of solid–solid transitions in TCNE at ambient pressure, reported between 292–326 K. In Table 5, the experimental and computed transition properties are compared. Enthalpy differences are found to be of the same order of magnitude, around 3.47 and 2.74 kJ/mol, respectively. Beyond the experimental analogues, the relative stabilities of the hypothetical forms are also considered in Figure 7. ID-9 and ID-13 are found to be the least stable over the studied temperature range, with free energies over 1 kJ/mol higher than the other structures. In contrast, ID-3 emerges as the most stable polymorph above 160 K—more stable than even the experimentally known cubic (ID-1) and monoclinic (ID-6) forms.

**Table 5:** Experimental and Calculated Properties for the solid–solid Transition from the ID-1 (or Cubic) Structure to the ID-6 (or Monoclinic) Structure^a^

Structure	*T*_ss_ (K)	Δ*H*_ss_ (kJ/mol)
Exp.	292,^60^ 318, 320, 326 ^58^	3.47 ^58^
ID-1/ID-6	322(9)	2.74(8)

a Numbers within parentheses indicate the statistical uncertainty in the last digit.

The enthalpic and entropic components of the Gibbs free energies, $\Delta G_{S_{1}\to S_{j}}=\Delta H_{S_{1}\to S_{j}}-T\Delta S_{S_{1}\to S_{j}}$ , are presented in Figure 8a and b. These decompositions highlight the driving forces behind the observed stability trends. ID-9 and ID-13 exhibit highly unfavorable enthalpic contributions, leading to their poor relative stability. The transition from ID-1 to ID-6 (experimental transition) is driven by entropy, with the entropic gain compensating for the enthalpic cost. The entropy-driven nature of enantiotropic temperature-mediated transitions is consistent with the findings of previous studies.^22,23,35,103^ Lastly, the transition from ID-1 to ID-3 exhibits the lowest enthalpic cost among all cases, which enables its high stability—particularly at temperatures below 300 K, where the entropic contributions across all forms are relatively similar.

**Figure 8:**
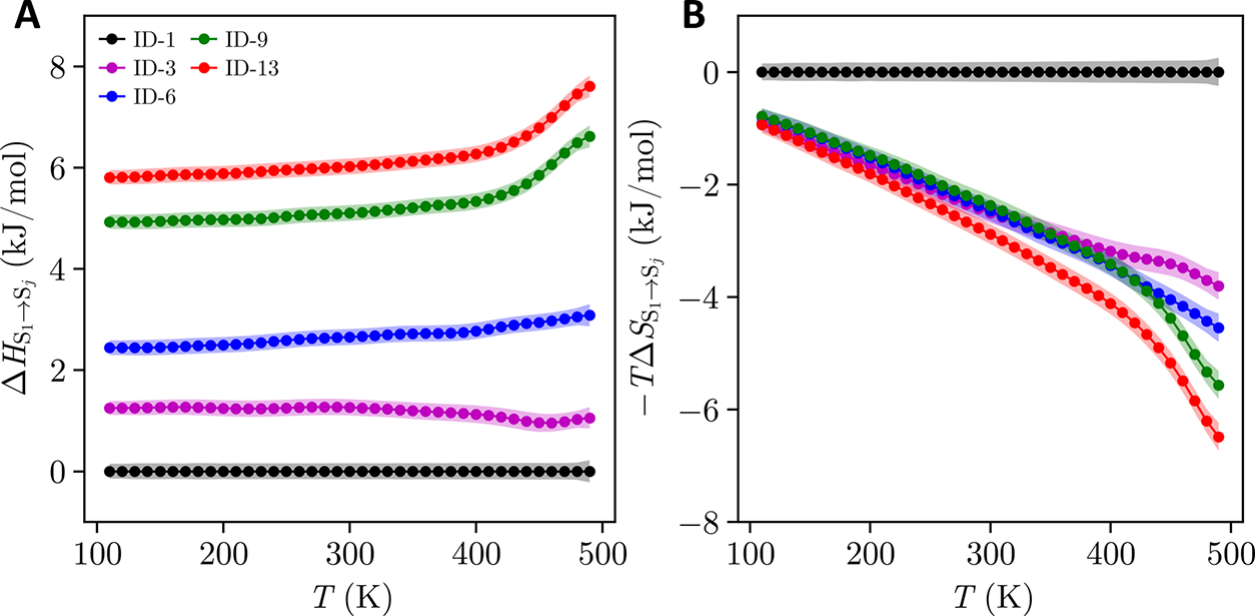
Decomposition of Gibbs free energy differences into (a) enthalpic and (b) entropic contributions for ID-1 (black), ID-3 (purple), ID-6 (blue), ID-9 (green), and ID-13 (red). The shaded regions indicate the associated uncertainties of the simulation results.

To further investigate the predicted trends in relative polymorphic stability, we reformulate the original PSCP decomposition at 350 K in terms of explicit intermolecular interactions, expressing the Helmholtz free energy difference as $\Delta A_{S_{1}\to S_{j}}\left(T_{\mathrm{ref}}\right)=\Delta A_{S_{1}\to S_{j}}^{\mathrm{vdW}}\left(T_{\mathrm{ref}}\right)+\Delta A_{S_{1}\to S_{j}}^{\mathrm{elec}}\left(T_{\mathrm{ref}}\right)+\Delta A_{S_{1}\to S_{j}}^{\mathrm{vol}}\left(T_{\mathrm{ref}}\right)$ . In this formulation, the van der Waals and electrostatic terms represent the interactions modulated along the alchemical path, while the volumetric term corresponds to $\Delta A_{{\mathrm{DWF}}_{1}\to \mathrm{WF}}-\Delta A_{{\mathrm{DWF}}_{j}\to \mathrm{WF}}$ . This interaction-based decomposition, shown in Table 6, offers a more nuanced view of the physical forces driving differences in relative polymorph stability. Methodological details on performing this decomposition are described in our previous work.^104^

**Table 6:** Decomposition of the Helmholtz Free Energy for PSCP-Modeled Solid–Solid Transitions at 350 K, into van der Waals, Electrostatic, and Volumetric Contributions^a^

Structure	$\Delta A_{S_{1}\to S_{j}}$ (kJ/mol)	$\Delta A_{S_{1}\to S_{j}}^{\mathrm{vdW}}$ (kJ/mol)	$\Delta A_{S_{1}\to S_{j}}^{\mathrm{elec}}$ (kJ/mol)	$\Delta A_{S_{1}\to S_{j}}^{\mathrm{vol}}$ (kJ/mol)
ID-1	0.00	0.00	0.00	0.00
ID-3	–1.66(9)	–6.41(7)	+3.80(5)	+0.955(1)
ID-6	–0.24(9)	–0.67(7)	+1.17(5)	–0.737(1)
ID-9	+2.33(9)	–0.52(7)	+3.63(4)	–0.773(1)
ID-13	+2.65(9)	+0.34(7)	+3.84(4)	–1.527(1)

a Numbers within parentheses indicate the statistical uncertainty in the last digit.

A high electrostatic penalty primarily accounts for the instability of ID-9 and ID-13, relative to ID-1. The corresponding electrostatic penalty for ID-6 is modest, enabling the transition from ID-1 through van der Waals and volumetric stabilization. Finally, the greater stability of ID-3, compared to ID-1, is driven exclusively by favorable van der Waals interactions, which offsets its electrostatic and volumetric terms. Notably, the enhanced van der Waals stabilization of ID-3 is 1 order of magnitude larger than that of the other forms. These results show that the MD model predicts a distinct behavior for ID-3 compared to the other structures, which appears to be the main reason for its predicted stability. A more in-depth investigation using alternative approaches, such as different force fields or QHA-based lattice dynamics, could help determine whether the stability of ID-3 reflects a true physical phenomenon or is an artifact of the computational model.

### 3.5 Solid–Liquid Transition Predictions

The capacity to describe fluid phases is a key advantage that MD has over LD. As such, MD simulations are also performed to compute the Gibbs free energy of liquid TCNE relative to each CSP-predicted structure between 350–490 K, as shown in Figure 9. The PSCP cycle, initially applied to solid–solid transitions, is supplemented with additional steps to analyze the solid–liquid transitions. Free energy contributions at each step of the PSCP protocol are listed in Table S3.

**Figure 9:**
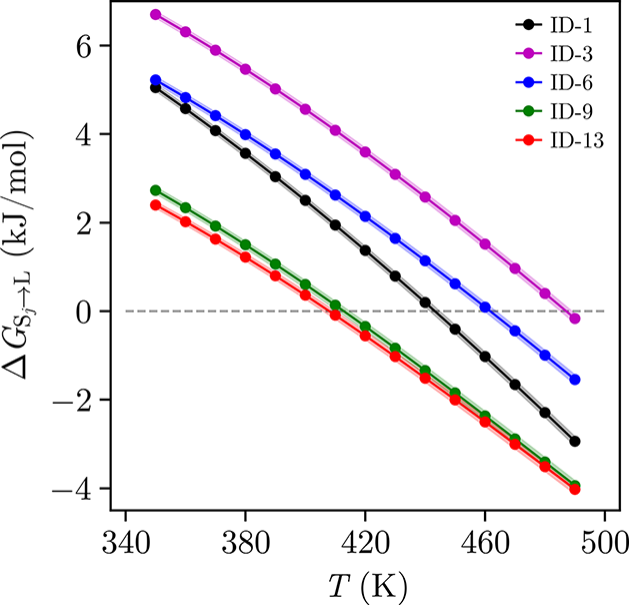
Difference in Gibbs free energy between liquid-phase TCNE and ID-1 (black), ID-3 (purple), ID-6 (blue), ID-9 (green), and ID-13 (red) as a function of temperature. The shaded regions indicate the associated uncertainties of the simulation results.

Based on the free energy analysis, the melting temperatures, along with the enthalpy and heat capacity differences between solid and liquid phases at that temperature, are calculated and summarized in Table 7. Experimentally, the monoclinic form of TCNE is observed to melt at 472 K.^105^ Our simulations of its corresponding crystalline form (ID-6) predict a transition at 464 K ± 3 K, an error of less than 2%. The calculated melting enthalpy of 24.57 kJ/mol ± 0.05 kJ/mol is also in excellent agreement with the experimental value of 24.92 kJ/mol.^105^ In addition to the monoclinic structure, we also evaluate the hypothetical melting properties of the other TCNE polymorphs. ID-3 shows the highest melting point (487 K, 28.22 kJ/mol), followed by ID-1 (443 K, 26.52 kJ/mol), ID-9 (413 K, 19.68 kJ/mol), and ID-13 (408 K, 18.74 kJ/mol). The results are consistent with the stability trend at elevated temperatures (ID-3 > ID-6 > ID-1 > ID-9 > ID-13), where more stable polymorphs exhibit higher melting temperature and enthalpy, reflecting stronger intermolecular interactions in the crystal lattice.

**Table 7:** Experimental and Calculated Melting Properties for Each Crystal Structure^a^

Structure	*T*_m_ (K)	Δ*H*_m_ (kJ/mol)	Δ*C*_*p*, m_ (J/mol/K)
Exp.^105^	472	24.92	
ID-1	443(3)	26.52(5)	51(2)
ID-3	487(3)	28.22(4)	54(2)
ID-6	464(3)	24.57(5)	46(2)
ID-9	413(2)	19.68(4)	62(3)
ID-13	408(2)	18.74(6)	62(3)

a Numbers within parentheses indicate the statistical uncertainty in the last digit.

### 3.6 Performance of the Free Energy Prediction Protocol

While good predictions of the finite temperature phase transitions are attained by our protocol, closer examination of the individual stages reveal opportunities for development.

The HA-LD prescreening step mitigates several limitations of progressing directly from a CSP_0 landscape to MD simulation.^41,43^ Not only does HA-LD provide an estimate of temperature-dependent entropic effects, which often drive changes in finite temperature stability, but it also enables the elimination of dynamically unstable crystals. Thus, through the HA-LD prescreening, one is afforded greater confidence that only the more thermodynamically relevant structures are brought forward for more expensive calculations. This is a cost-effective approach for reducing the well-known CSP_0 overprediction problem.^24,106–108^

With that said, the reliability of HA-LD for assessing thermodynamic relevance should also be scrutinized. LD-based methods are sensitive to numerical inaccuracies in the preceding lattice energy minimization—failing to converge tightly to a genuine static lattice minimum can lead to spurious assessments of dynamical instability using LD.^109^ Among the 30 unstable structures exhibiting imaginary frequencies, 25 are due to this type of insufficient numerical convergence. In our current work, we discard these structures, but one could in principle include an additional minimization stage, for example by using analytical Hessian matrices, to further converge these structures prior to HA-LD. The remaining 5 structures are indeed unstable as the corresponding dynamical matrix exhibits at least one negative eigenvalue for at least one reciprocal space vector.

From a theoretical standpoint, the assumptions that underpin the HA-LD model appear reasonable for TCNE but are not applicable to all systems, with changes in cell volume^33^ and/or greater anharmonicity being associated with more-flexible molecules.^28,52^ It is difficult to deduce (*a priori*) when anharmonicity is significant but the extent of volumetric changes could potentially be inferred from the subsequent MD results. Where large changes are observed, it would be prudent to advance more structures for MD refinement.

Following the prescreening, application of the PSCP method to finite-temperature free energy analysis was found to be fairly successful. Despite only minor system-specific parametrization, the MD simulations were able to reproduce most experimental observations of solid–solid and solid–liquid phase-transition behavior, within an acceptable level of accuracy. The MD-predicted cubic/monoclinic enantiotropic transition temperature is in better agreement with experiments than that predicted with HA-LD. This corroborates our suggested hierarchy of finite temperature analysis methodologies, wherein the inclusion of anharmonic and volumetric effects in MD bolsters its accuracy as a final ranking tool. However, the odd prediction of ID-3 stability could point to limitations in state-of-the-art MD models. Whether this result is a genuine physical effect or an artifact of the MD model remains uncertain and warrants further investigation, as discussed later.

In a broader context, the ability to predict a range of melting properties using MD creates an opportunity to extend the *in silico* workflow with other synergistic modeling tools. For example, the three melting properties predicted in this work (*T*_m_, Δ*H*_m_, Δ*C*_*p*, m_) can be utilized in solubility prediction with equations of state,^102,110,111^ or activity coefficient models.^112,113^ This is possible because, unlike HA-LD methods that are restricted to crystalline phases, MD can directly access the thermodynamics of disordered fluids, enabling consistent evaluation of liquid free energies alongside solids.

However, in applying these predictions, it is also important to remain cognizant of the uncertainties (statistical or otherwise) associated with these values. The confidence bands in the free energy profiles represent statistical and sampling uncertainties from the MD/MBAR analysis and do not include systematic errors associated with force field and charge-model parametrization. Because Δ*G* is obtained as the difference between two absolute free energies, similar biases in the individual phases may partially cancel, leading to apparently narrower confidence bands.^102^ When such correlations are weak or of opposite sign, this cancellation diminishes, resulting in broader 95% intervals. In all cases, the actual uncertainties may exceed the reported values, since systematic model errors are not captured by the statistical analysis. At higher temperatures, enhanced anharmonicity and sampling variance further increase these deviations. In our simulations, an uncertainty of 0.09 kJ/mol in $\Delta G_{S_{1}\to S_{6}}$ resulted in a 9 K uncertainty in the predicted ID-1/ID-6 transition temperature, illustrating the strong dependence of *T*_ss_ on the free energy differences, consistent with previous studies showing that phase transition temperatures can vary substantially in response to small changes in the relative free energies of the competing phases.^114^ Those authors also noted that small correlated errors in enthalpy and entropy can either compensate or amplify deviations in free energy differences, occasionally leading to apparent agreement with experiment through error cancellation.

The stated intent of this work was to introduce a workflow that mitigates the cost constraints associated with MD analysis. To this end, our use of a modified OPLS force field proved adequate, but this should not be viewed as an endorsement of this force field in all future applications of the proposed workflow. As demonstrated here, the suitability of any force field to model the system of interest should be established with available data. For this purpose, the use of an orthogonal free energy analysis technique (HA-LD) can provide a convenient benchmark to evaluate the MD model, a strategy which is especially valuable under blind conditions.

Ultimately, the accuracy of MD models remains an important obstacle to the widespread adoption of CSP → MD workflows such as the one proposed here. Even with careful parametrization/validation, the classical point-charge force fields commonly applied in MD simulations will likely struggle to juggle the requirements of accurately modeling diverse organic molecules in both solid and liquid phases. One potential advancement could be the introduction of distributed multipole electrostatics into the MD model. This is appealing since multipoles improve the description of directional interactions in crystals (e.g., hydrogen bonds)^115,116^ and would also permit greater consistency in the energy model across the CSP, LD, and MD stages. MD packages like DL_MULTI^90,117^ /DL_POLY^118^ support such capabilities and have been employed to simulate aqueous solutions,^119^ organic solids,^120–122^ and molecular clusters in solution.^120^ However, the development of multipole-based MD force fields^123,124^ remains limited in comparison to their point-charge counterparts. Besides the addition of higher-ranked multipoles, another avenue is the application of the existing workflow in conjunction with machine learning (ML) potentials either via foundation models or via tailored models.^125–129^ However, the applicability of ML models for the proposed use cases has not been well studied and warrants further investigation.

In any case, employing a more sophisticated model (multipole- or ML-based) in our MD stage will add considerable cost to the MD simulation; this further underscores the need for applying (relatively) inexpensive prescreening. It is hoped that using these more accurate models in conjunction with other MD techniques (e.g., metadynamics^41,43,106,107^ ), the thermodynamic and kinetic factors governing solid phase transitions may be elucidated with greater reliability. This could serve as an alternative model to help resolve the ambiguous stability predictions observed for the ID-3 structure, for example.

## 4 Conclusions

In this work, we have proposed a general three-stage computational workflow to assess the thermodynamic stability of molecular crystals at finite temperature conditions. The method integrates: (i) zeroth-order crystal structure prediction (CSP) for candidate generation; (ii) harmonic approximation-based lattice dynamics (HA-LD) for coarse prescreening of finite temperature behavior; and finally (iii) molecular dynamics (MD) simulations for rigorous assessment of finite temperature phase stability.

The proposed workflow was applied on an organic molecule, tetracyanoethylene (TCNE). In the spirit of a “blind test”, we strived to use as little prior knowledge of TCNE polymorphs as possible in the decision-making between stages. Under these conditions, the known forms of TCNE at ambient conditions were successfully generated and advanced through the protocol as thermodynamically competitive candidates. Ultimately, the important solid–solid and solid–liquid phase behavior of crystalline TCNE were predicted with good accuracy, and one hypothetical structure exhibited unanticipated thermodynamic stability relative to the known forms.

To our knowledge, this is the first instance of LD and MD being used in an integrated workflow. The favorable balance of cost and accuracy offered by HA-LD makes it an ideal choice for prescreening CSP candidates prior to more-rigorous MD modeling. This prescreening is a vital bridging step. Not only does it reduce the likelihood of erroneously rejecting putative structures between the static and dynamical analyses, it also offers an orthogonal technique to validate/corroborate the findings from MD. Overall, our workflow combines HA-LD and MD in a complementary manner, leveraging the strengths of each method while compensating for their respective shortcomings.

Such a multistage workflow represents a natural extension of the zeroth-order CSP paradigm, and will be essential for enabling more accurate and sophisticated finite temperature analysis techniques. Future work should focus on exploring these developments in the ultimate stage, including improvements to MD force field accuracy and strategies to investigate the kinetics governing phase stability. This would further strengthen the workflow’s predictive power and broaden the scope of wholly *in silico* materials discovery and process design.

## Supplementary Material



## Data Availability

The data supporting this article have been included as part of the Supporting Information. The data analysis scripts are available at github.com/MaginnGroup/CSP-LD-MD-Paper.

## Notes

Force field parameters used in molecular dynamics simulations; relative Helmholtz free energy landscape obtained from lattice dynamics calculations; temperature-dependent density and enthalpy profiles of solid phases obtained from molecular dynamics simulations; angular and dihedral distribution profiles of solid phases obtained from molecular dynamics simulations; free energy contributions from the PSCP method used in solid–liquid and solid–solid transition analyses (PDF)
