# Supplementary material for: Assessing Polymorph Stability and Phase Transitions at Finite Temperature: Integrating Crystal Structure Prediction, Lattice Dynamics, and Molecular Dynamics
Source: J Chem Theory Comput. 2025 Nov 19;21(23):12197–213. doi: 10.1021/acs.jctc.5c01387 (PMC12874377; doi:10.1021/acs.jctc.5c01387)
Supplement: Supplementary file 1 [file ct5c01387_si_001.pdf]

Supplementary Material

Assessing Polymorph Stability and Phase Transitions at Finite Temperature: Integrating Crystal Structure Prediction, Lattice Dynamics, and Molecular Dynamics

Gabriela B. Correa,[†,‡] Stefanos Konstantinopoulos,[¶] Benjamin I. Tan,[¶] Yong Zhang,[†] Frederico W. Tavares,[‡] Claire S. Adjiman,[¶] and Edward J. Maginn[*,†]

†*Department of Chemical and Biomolecular Engineering, University of Notre Dame, Notre Dame, Indiana 46556, USA*

‡*Chemical Engineering Program, Universidade Federal do Rio de Janeiro, Rio de Janeiro 21941-909, Brazil*

¶*Department of Chemical Engineering, Sargent Centre for Process Systems Engineering, Imperial College London, London SW7 2AZ, United Kingdom*

E-mail: ed@nd.edu

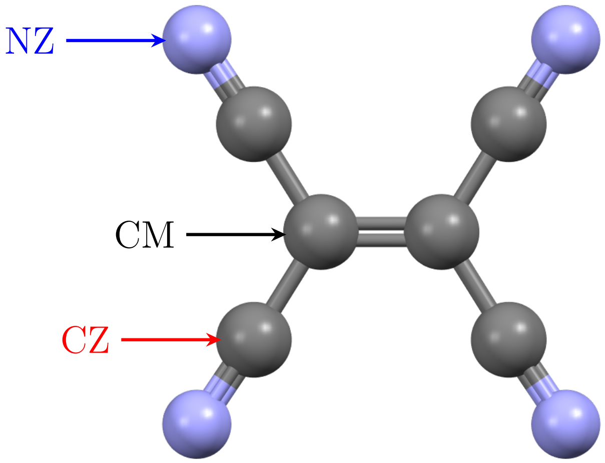

Figure S1: Tetracyanoethylene (TCNE, C_6N_4) molecule. The atom types used in MD simulations (NZ, CZ, and CM) are also labeled.

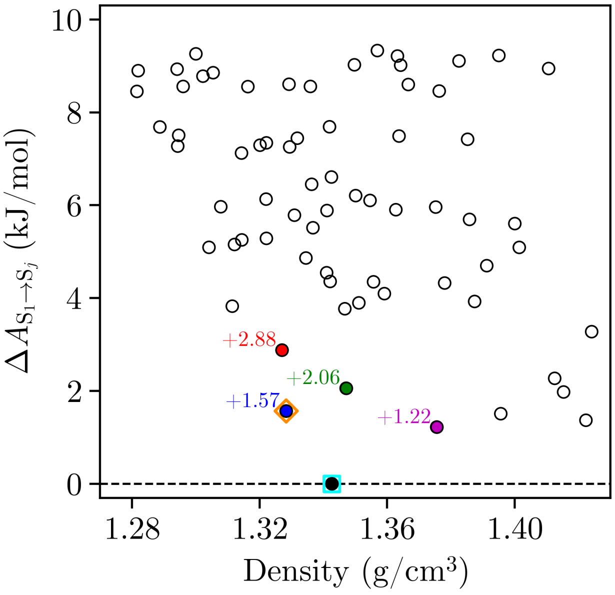

Figure S2: Relative Helmholtz free energy landscape for the 70 CSP-predicted structures with positive phonon frequencies at 0 K. CSP-predicted structures are given by unfilled black circles, while matches to the cubic and monoclinic forms are indicated by the unfilled cyan square and unfilled orange diamond, respectively. ID-1 (black), ID-3 (purple), ID-6 (blue), ID-9 (green), and ID-13 (red) are denoted by filled circles and labeled by their relative energies, compared to ID-1 (dashed black line).

Table S1: Parameters for TCNE used in MD simulations. Derived from the all-atom OPLS/2020 force field, with partial charges (q) and equilibrium intramolecular bond angles (θ_0) refined using B3LYP/6-311++G(d,p) quantum chemical calculations.

Nonbonded parameters			
	NZ	CZ	CM
σ (Å)	3.20	3.30	3.55
ϵ (kJ/mol)	0.711	0.276	0.318
q (esu)	−0.359	0.405	−0.092
Bond parameters $K(r-r_0)^2$			
	CZ-NZ	CZ-CM	CM-CM
r_0 (Å)	1.160	1.426	1.340
K (kJ/mol/Å^2)	2720	1674	2297
Angle parameters $K(\theta-\theta_0)^2$			
	CM-CZ-NZ	CZ-CM-CZ	CM-CM-CZ
θ_0 (deg)	178.9	116.8	121.6
K (kJ/mol/rad^2)	292.88	292.88	292.88
Dihedral parameters $\sum_i K_i/2(1 \pm \cos(i\psi))$			
	X-CM-CZ-X	CZ-CM-CM-CZ	
K_1 (kJ/mol)	0.0	0.0	
K_2 (kJ/mol)	0.0	58.58	
K_3 (kJ/mol)	0.0	0.0	

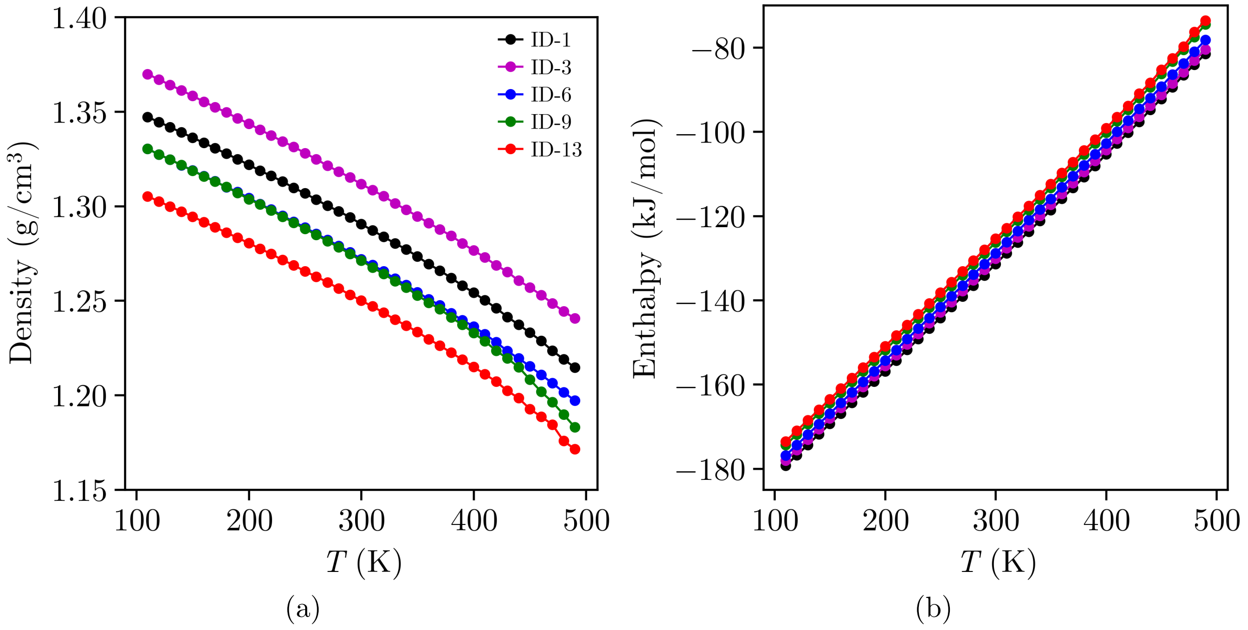

Figure S3: (a) Density and (b) enthalpy profiles of ID-1 (black), ID-3 (purple), ID-6 (blue), ID-9 (green), and ID-13 (red) over 110–490 K. The data are obtained from NPT ensemble simulations run for 5 ns, with properties sampled every 200 fs. The smooth decrease in density and increase in enthalpy with temperature indicate structural stability, with no significant changes in the solids or direct melting observed throughout the simulation range.

Table S2: Free energy contributions in kJ/mol from the PSCP method at 350 K for solid–solid transitions. In the $\mathrm{S}_j \to \mathrm{DWF}_j$ step, well depths of the Gaussian potential are tested at two values, 20.92 and 41.84 kJ/mol, and their average is used in the calculations. Numbers in parentheses indicate the statistical uncertainty in the last digit.

Structure	$\Delta A_{\mathrm{S}_j \to \mathrm{DWF}_j}$ 20.92 kJ/mol	$\Delta A_{\mathrm{S}_j \to \mathrm{DWF}_j}$ 41.84 kJ/mol	$\Delta A_{\mathrm{S}_j \to \mathrm{DWF}_j}$ average	$\Delta A_{\mathrm{DWF}_j \to \mathrm{WF}}$	$\Delta A_{\mathrm{S}_1 \to \mathrm{S}_j}$	$P\Delta V_{\mathrm{S}_1 \to \mathrm{S}_j}$	$\Delta G_{\mathrm{S}_1 \to \mathrm{S}_j}$
ID-1	+165.71(9)	+165.75(9)	+165.73(9)	−5.401(1)	0	0	0
ID-3	+168.33(9)	+168.36(9)	+168.34(9)	−6.356(1)	−1.66(9)	-2×10^{-4}	−1.66(9)
ID-6	+165.24(9)	+165.22(9)	+165.23(9)	−4.664(1)	−0.24(9)	$+2 \times 10^{-4}$	−0.24(9)
ID-9	+162.64(7)	+162.62(7)	+162.63(7)	−4.628(1)	+2.33(9)	$+2 \times 10^{-4}$	+2.33(9)
ID-13	+161.55(6)	+161.56(6)	+161.55(9)	−3.874(1)	+2.65(9)	$+3 \times 10^{-4}$	+2.65(9)

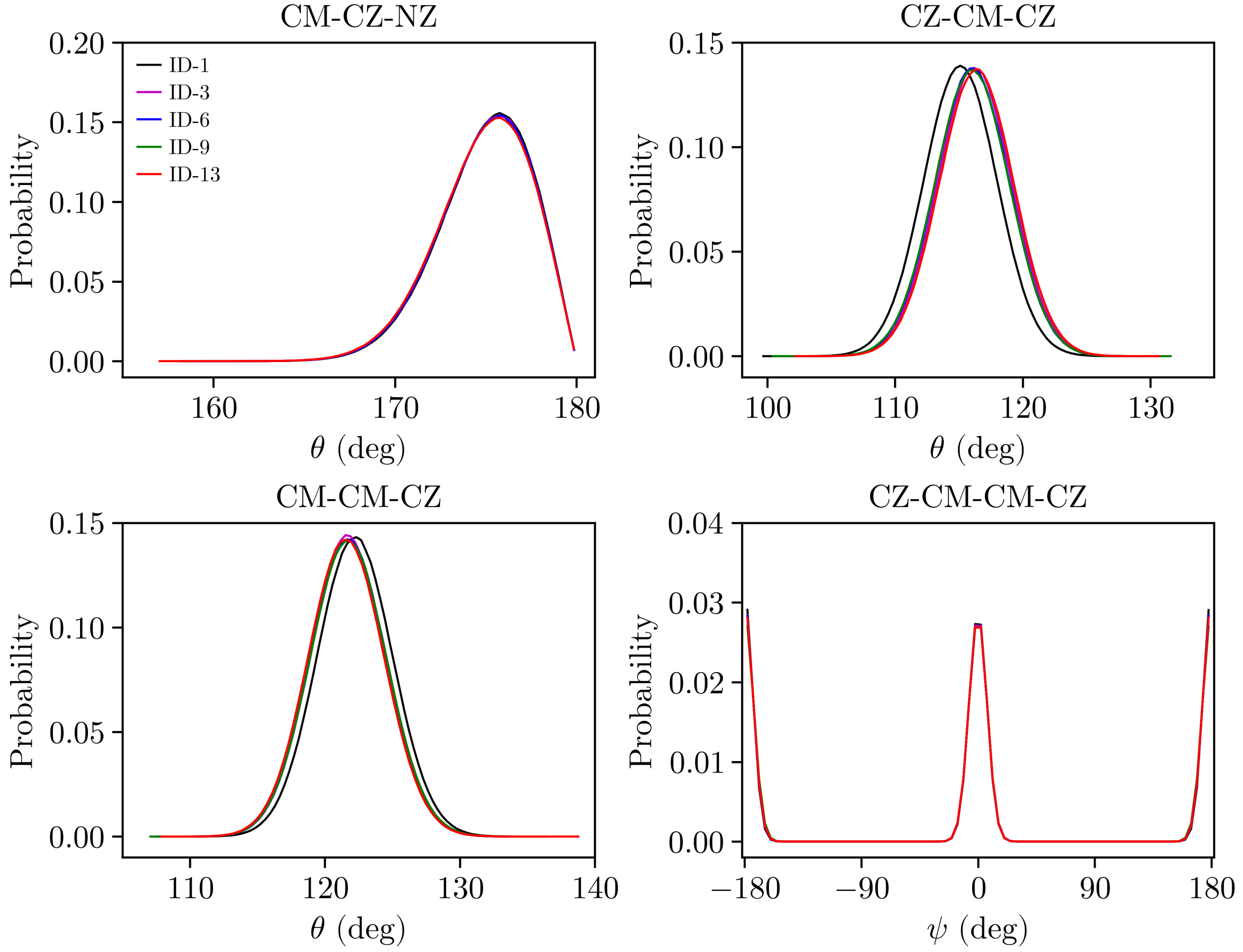

Figure S4: Angular and dihedral profiles of ID-1 (black), ID-3 (purple), ID-6 (blue), ID-9 (green), and ID-13 (red), based on atom types defined in Figure S.1 and Table S.1. The data are obtained from NVT ensemble simulations at 295 K run on equilibrated structures for 5 ns, with configurations sampled every 200 fs. TCNE remains planar, with dihedral angles close to 180°. ID-1 (cubic) shows slightly shifted bond angles compared to the other forms.

Table S3: Free energy contributions in kJ/mol from the PSCP method at 350 K for solid–liquid transitions. Numbers in parentheses indicate the statistical uncertainty in the last digit.

Structure	$\Delta A_{\mathrm{S}_j \to \mathrm{DWF}_j} + \Delta A_{\mathrm{DWF}_j \to \mathrm{WF}}$	$\Delta A_{\mathrm{WF} \to \mathrm{L}}$	$\Delta A_{\mathrm{S}_j \to \mathrm{L}}$	$P\Delta V_{\mathrm{S}_j \to \mathrm{L}}$	$\Delta G_{\mathrm{S}_j \to \mathrm{L}}$
ID-1	+160.33(9)		+5.05(9)	$+1 \times 10^{-3}$	+5.05(9)
ID-3	+161.98(9)		+6.70(9)	$+2 \times 10^{-3}$	+6.70(9)
ID-6	+160.57(9)	−155.28(3)	+5.29(9)	$+1 \times 10^{-3}$	+5.29(9)
ID-9	+158.00(7)		+2.72(7)	$+1 \times 10^{-3}$	+2.72(7)
ID-13	+157.68(9)		+2.40(9)	$+1 \times 10^{-3}$	+2.40(9)
